# Fully Automated Serum LC-MS/MS Platform and Pediatric Reference Intervals for Organic Acids, Amino Acids, and Acylcarnitines in Children (Ages 0–6 Years): Toward Quantitative Diagnosis of Inborn Errors of Metabolism

**DOI:** 10.3390/diagnostics16060911

**Published:** 2026-03-19

**Authors:** Yasushi Ueyanagi, Daiki Setoyama, Tsuyoshi Nakanishi, Yuichi Mushimoto, Vlad Tocan, Hironori Kobayashi, Miki Matsui, Shinya Matsumoto, Akiyoshi Fujishima, Taeko Hotta, Ayumi Sakata, Yuya Kunisaki

**Affiliations:** 1 Department of Clinical Laboratory, Kyushu University Hospital, Fukuoka 812-8582, Japan; ueyanagi.yasushi.964@m.kyushu-u.ac.jp (Y.U.); matsumoto.shinya.544@m.kyushu-u.ac.jp (S.M.); fujishima.akiyoshi.990@m.kyushu-u.ac.jp (A.F.); sakata.ayumi.791@m.kyushu-u.ac.jp (A.S.); 2 Shimadzu Corporation, Kyoto 604-8511, Japan; naka@shimadzu.co.jp; 3 Department of Pediatrics, Graduate School of Medical Sciences, Kyushu University, Fukuoka 812-8582, Japan; mushimoto.yuichi.831@m.kyushu-u.ac.jp (Y.M.); tocan.vlad.875@m.kyushu-u.ac.jp (V.T.); 4 Clinical Laboratory Division, Shimane University Hospital, Izumo 693-8501, Japan; 5 Department of Pediatrics, Hyogo College of Medicine, Nishinomiya 663-8501, Japan; matsu.m.0878@gmail.com; 6 Department of Clinical Laboratory, International University of Health and Welfare Narita Hospital, Narita 286-8520, Japan; hotta-taeko-uz@ihwg.jp; 7 Department of Clinical Chemistry and Laboratory Medicine, Graduate School of Medical Sciences, Kyushu University, Fukuoka 812-8582, Japan

**Keywords:** liquid chromatography–tandem mass spectrometry (LC–MS/MS), inborn errors of metabolism (IEMs), organic acids, amino acids, acylcarnitines, pediatric reference intervals

## Abstract

**Background/Objectives:** Conventional diagnosis of inborn errors of metabolism (IEMs) requires multiple specimen types—urine organic acids, plasma amino acids, and serum acylcarnitines—analyzed on distinct analytical platforms. This multi-assay approach is labor-intensive and limits timely clinical decision making. We aimed to develop a fully automated serum-based LC–MS/MS platform for integrated quantitative metabolite profiling and to establish pediatric reference intervals (RIs) to support diagnostic interpretation. **Methods:** A fully automated LC–MS/MS system integrated with the CLAM-2030 automated pretreatment module was developed to enable simultaneous quantification of 25 organic acids, 8 amino acids, and 21 acylcarnitines. Analytical performance was assessed for linearity, limits of detection and quantification, precision and accuracy. Serum samples from 296 non-IEM children aged 0–6 years were analyzed to establish pediatric RIs using Box–Cox transformation and Gaussian modeling. Clinical utility was evaluated in sera from 89 patients diagnosed with IEM using z-score-based logistic regression models. **Results:** The method demonstrated excellent performance, with linearity (r^2^ > 0.99) across calibration ranges, limits of detection and quantification defined by signal-to-noise ratios > 3 and >10, and intra- and inter-assay precision < 15% CV for all 54 analytes. Twenty-one analytes met the acceptance criterion of ±20% accuracy at all quality-control levels. Pediatric RIs provided a quantitative framework for interpreting the metabolic abnormalities. In IEM patients, disease-specific metabolites were consistently outside the established ranges, and z-score-based logistic regression models successfully distinguished major IEM categories, including organic acidemias and long-chain fatty acid oxidation disorders. **Conclusions:** This fully automated, serum-based LC–MS/MS platform provides a clinically practical and quantitative framework for integrated metabolic profiling using pediatric RIs, supporting diagnosis and monitoring of IEMs in pediatric settings.

## 1. Introduction

Inborn errors of metabolism (IEMs) are genetic disorders that disrupt intermediary metabolic pathways, leading to the accumulation or deficiency of specific metabolites [1]. Collectively, IEMs affect approximately 1 in 2000 newborns worldwide, and their early and accurate evaluation is critical for clinical management, prognosis, and therapeutic monitoring [2,3].

Conventionally, differential diagnosis of IEMs requires the combination of analyses with multiple specimen types—urinary organic acids [4,5,6,7], plasma amino acids [8,9,10], and serum acylcarnitines [11]—using distinct analytical platforms. While this multi-specimen approach remains the diagnostic standard, it is labor-intensive, time-consuming, and unsuitable for rapid or repeated monitoring in clinical settings. The need for multiple sample collections can be particularly challenging in neonates and infants, where specimen volume and timing are often limiting factors [1,12,13].

Recent advances in liquid chromatography-tandem mass spectrometry (LC-MS/MS) have enabled simultaneous quantification of structurally diverse metabolites with high sensitivity and specificity [14]. Building on this, our previous study demonstrated that a fully automated LC–MS/MS system integrating the CLAM-2030 pretreatment module could quantify organic acids in serum samples within one hour, providing a clinically feasible alternative to gas chromatography-mass spectrometry [15]. Extending this approach to include amino acids and acylcarnitines within a single serum-based analytical framework would enable a comprehensive assessment of mitochondrial, amino acid and fatty acid oxidation pathways—key domains affected in many organic acidemias and fatty acid oxidation disorders.

Establishing pediatric reference intervals (RIs) for these serum metabolites is essential to translate such multiplex measurements into quantitative clinical interpretation. Pediatric metabolic profiles differ markedly from those of adults because of developmental changes in enzyme activity, nutritional status, and organ maturation [16]. Defining age-specific RIs, particularly in early children (0–6 years), allows objective quantification of metabolic deviations and facilitates longitudinal evaluation of disease activity or treatment response in patients with IEMs [17,18].

Moreover, the accumulation of large-scale, standardized metabolic data generated by such a fully automated platform provides an opportunity for data-driven diagnostic support. Quantitative deviations from pediatric RIs, when expressed as standardized z-scores, can be analyzed using machine-learning algorithms to identify disease-specific metabolic patterns. This integration of automation and computational modeling has the potential to transform metabolite profiling from qualitative pattern recognition to quantitative, reproducible diagnosis and classification of IEMs.

This study was designed to support diagnostic differentiation of inborn errors of metabolism included in current newborn screening programs in Japan (Supplemental Table S7).

In this study, we report the development and validation of a fully automated LC–MS/MS platform capable of simultaneously quantifying serum organic acids, amino acids, and acylcarnitines. We further establish pediatric (0–6 years) serum RIs for 54 metabolites and evaluate their clinical utility in patients with confirmed IEMs. Finally, we propose that quantitative, data-driven approaches may further advance precision diagnostics for inherited metabolic diseases.

## 2. Materials and Methods

### 2.1. Reagents

Ultrapure water, formic acid, methanol, and acetonitrile (FUJIFILM Wako Pure Chemical Corporation, Osaka, Japan) were used. 3-Nitrophenylhydrazine (3-NPH) and N-(3-dimethylaminopropyl)-N′-ethylcarbodiimide (EDC) hydrochloride were obtained from Sigma-Aldrich (St. Louis, MO, USA). Analytical and internal standards are listed in Supplemental Table S1.

### 2.2. Stock Solutions and QC Samples

Internal standards followed Table S1 (amino acids/acylcarnitines from NeoSMAAT kit (Sekisui Medical Co., Tokyo, Japan). Other analytes were dissolved in water and stored at −80 °C. Quality-control (QC) sera at three concentration levels—low, medium, and high quality control (LQC/MQC/HQC)—were prepared by spiking pooled serum and stored at −80 °C.

### 2.3. Sample Collection

All procedures were approved by the Kyushu University Ethics Committee (No. 22138, approved on 28 November 2024).

**Children without inborn errors of metabolism**.

We collected 296 residual serum samples from children aged 0–6 years who visited the outpatient clinic of the Department of Pediatrics, Kyushu University Hospital.

Inclusion criteria: creatinine < 0.48 mg/dL; AST < 68 U/L; no visible hemolysis.

Exclusion criteria (prespecified):

• Confirmed or suspected inborn errors of metabolism.
• Acute-phase conditions or immediately post-infectious state.
• Malignant disease.
• Trisomy 21.
• Ethnicity control: both parents self-identified as Japanese (to minimize inter-ethnic variability when establishing pediatric serum reference intervals).
• Medications or therapies affecting metabolites: ongoing valproate treatment; carnitine supplementation; post-organ transplantation receiving immunosuppressive therapy; treatment with intravenous immunoglobulin preparations.

Age and sex distributions are summarized in Table S2.

**Patients with IEMs.**

Stored serum samples from 89 biochemically diagnosed, independent cases were included (Kyushu University, *n* = 5; Shimane University, *n* = 84), covering propionic acidemia (OMIM#606054), methylmalonic acidemia (OMIM#251000), isovaleric acidemia (OMIM#243500), 3-methylcrotonyl-CoA carboxylase deficiency (OMIM#210200), glutaric acidemia type II (OMIM#231680), medium-chain acyl-CoA dehydrogenase deficiency (OMIM#201450), very-long-chain acyl-CoA dehydrogenase deficiency (OMIM#201475), carnitine palmitoyltransferase II deficiency (OMIM#255110), mitochondrial trifunctional protein deficiency (OMIM#609015, #620300), phenylketonuria (OMIM#262600), and ornithine transcarbamylase deficiency (OMIM#311250). Case distribution is shown in Table S3.

### 2.4. Automated Sample Preparation

Protein precipitation and 3-NPH derivatization were performed on a CLAM™-2030 automated pretreatment system (Shimadzu, Kyoto, Japan) as previously described [15]. Pretreated extracts were analyzed on an LCMS™-8050 (Shimadzu, Kyoto, Japan) with a 5 µL injection volume.

### 2.5. LC–MS/MS Conditions

Chromatography was performed using a Shim-pack Scepter HD-C18-80 (150 × 2.1 mm, 3 µm) column (Shimadzu GLC, Kyoto, Japan). Mobile phases were (A) water with 0.1% formic acid and (B) acetonitrile with 0.1% formic acid, at 0.35 mL/min, with a 24-min gradient (16–70% B over 0–18 min, 95% B wash, and re-equilibration to 16% B). The column oven was maintained at 40 °C. Detection was performed by ESI-MRM with the following settings: nebulizing gas 3.0 L/min; drying gas 15.0 L/min; desolvation line 250 °C; heat block 400 °C; collision gas 230 kPa. Dwell times (22–163 ms) ensured ≥20 points per peak. MRM transitions for all 3-NPH-derivatized analytes are listed in Table S4. Four acylcarnitines (C16:1, C16OH, C18:1, C18:1OH) were empirically assigned using a mitochondrial trifunctional protein deficiency (OMIM #609015, #620300) serum, monitoring a characteristic product ion at *m*/*z* 220.1.

### 2.6. Method Validation

**Linearity**: Linearity was evaluated for 50 analytes, excluding four compounds without available reference standards (AC_C16:1, AC_C16OH, AC_C18:1, and AC_C18:1OH). Calibration samples were prepared by serial two-fold dilutions of the mixed standard solution to generate ten concentration levels. For analytes with internal standards, calibration curves were constructed from the relationship between the peak-area ratio (analyte/internal standard) and concentration; for those without internal standards, absolute peak areas were used. Linearity was considered acceptable when the coefficient of determination (r^2^) exceeded 0.99.

**Sensitivity**: Limit of detection (LOD) and quantification (LOQ) were defined at signal-to-noise ratios greater than 3 and 10, respectively, using five additional dilutions below the lowest calibrator.

**Accuracy and Imprecision**: For 34 analytes with internal standards, single-point calibration was applied with an accuracy acceptance was ±20%. Imprecision was evaluated at LQC/MQC/HQC (intra-/inter-day, *n* = 5 each), with CV < 15% as the criterion. For analytes without internal standards, variability was assessed from peak areas.

### 2.7. Data Analysis and Statistical Processing

All LC–MS/MS data were processed using LabSolutions software (version 5.97 SP1, Shimadzu Corporation, Kyoto, Japan). Peak areas were used as indicators of signal intensity. For pyruvic acid and 3-methylglutaconic acid, two peaks were observed and their summed values were used. Quantitative analytes below LOD were imputed with LOD values; others were set to a fixed area of 2000.

**Reference intervals (RIs).**

Pediatric RIs were established according to the harmonized protocol of the IFCC Committee on Reference Intervals and Decision Limits (C-RIDL) [19], which allows parametric or Box–Cox-transformed estimation after normalization and outlier exclusion. A parametric approach with Box–Cox transformation was used to approximate normality. After transformation, observations exceeding ±2.81 SD were excluded as outliers, and RI limits were derived by back-transforming ±1.96 SD to the original scale. When more than 2.5% of the control population fell below LOD, only the upper limit was reported. This C-RIDL-based procedure ensures international comparability and harmonization of RI estimates across institutions.

**Z-score calculation and multivariate analysis.**

Z-scores were computed after log_10_ transformation of quantitative values or peak areas, referenced against the mean and SD of the non-IEM children group (*N* = 296). For analytes with fewer than 100 valid data points, SD was adjusted so that a measurement corresponding to five times the LOD yielded a Z-score of 2.0. Multinomial logistic regression (MLR) models were constructed to evaluate whether diseases with related metabolic pathways could be discriminated from one another. The explanatory variables consisted of Z-scores representing standardized metabolite concentrations. For each disease category, a model was built using all metabolites known to be diagnostically relevant to that specific disorder, thereby including all established biomarker rather than restricting the model to only statistically significant variables. All modeling and performance evaluations were performed in R (version 4.4.0).

## 3. Results

### 3.1. Fully Automated Serum LC-MS/MS Platform

**Figure 1:**
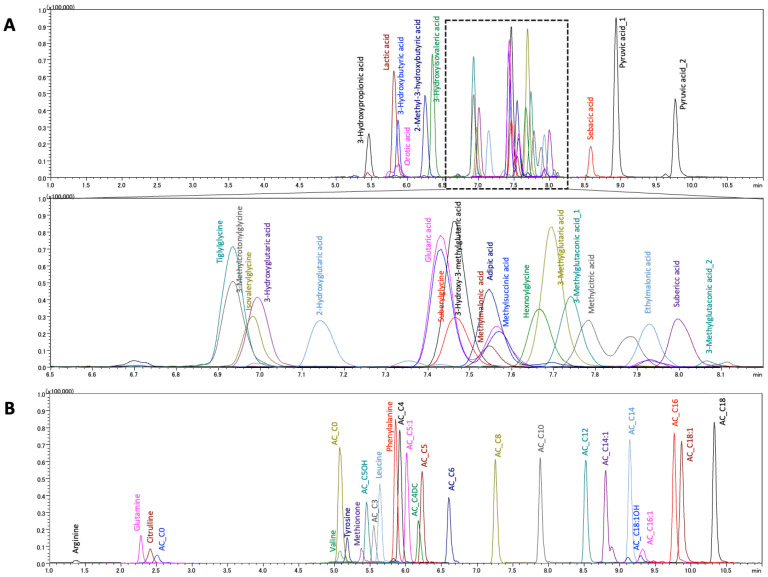
MRM chromatograms obtained using the developed LC-MS/MS method. (**A**) Chromatogram of organic acids analyzed using the standard solution. (**B**) Chromatogram of amino acids and acylcarnitines analyzed using the standard solution. For AC_C16:1, C16OH, C18:1, C18:1OH, standards were not available; therefore, retention times and MRM transitions were confirmed using a serum sample from a patient with mitochondrial trifunctional protein deficiency. Representative chromatograms shown here were obtained from normal serum.

Building on our previous report, we refined a fully automated workflow integrating on-instrument sample preparation (CLAM-2030) with LC–MS/MS [15] to enable simultaneous quantification of 25 organic acids, 8 amino acids, and 21 acylcarnitines from a single serum specimen. The total analysis time was within 60 min per sample, supporting routine implementation in clinical laboratories. All analytes were derivatized with 3-nitrophenylhydrazine (3-NPH) and monitored in multiple reaction monitoring (MRM) mode. For four acylcarnitines (C16:1, C16OH, C18:1, and C18:1OH), MRM transitions were determined empirically using serum from a patient with mitochondrial trifunctional protein (TFP) deficiency, with precursor ions yielding the characteristic fragment at *m*/*z* 220.1. A complete list of transitions is provided in Table S4, and representative ion chromatograms are shown in Figure 1. Theses refinements extend the analytical scope from organic acids alone to a multidimensional metabolic panel encompassing mitochondrial, amino acid, and fatty acid oxidation pathways, enabling comprehensive metabolic profiling from serum.

### 3.2. Linearity, Sensitivity, Accuracy and Imprecision

Calibration curves were prepared for 50 analytes with reference standards. Acceptable linearity (r^2^ > 0.99) was confirmed across eight or more concentration levels for all analytes (Table 1). Limits of detection (LOD) and quantification (LOQ) were determined by serial dilutions (signal-to-noise ratios of >3 and >10, respectively). Accuracy was assessed for 34 analytes with internal standards using single-point calibration: 21 analytes showed deviations within ±20% at all quality-control levels. Imprecision was <15% coefficient of variation (CV) for all 54 analytes in both intra- and inter-day assessments, consistent with ICH M10 and FDA bioanalytical guidelines [20,21] (Table 2). These results confirm the analytical robustness and quantitative reliability of the automated LC-MS/MS platform for clinical application.

### 3.3. Pediatric Serum RIs (0–6 Years)

To enable quantitative interpretation of serum metabolite profiles, pediatric RIs were established for 54 analytes using serum samples from 296 non-IEM children aged 0–6 years. Descriptive statistics of this non-IEM cohort are provided in Table S5.

Reference intervals were calculated according to the statistical approach described in Section 2.7, following the harmonized IFCC Committee on Reference Intervals and Decision Limits (C-RIDL) protocol [19]. The resulting pediatric serum RIs are summarized in Table 3. For analytes in which more than 2.5% of the control population had concentrations below the limit of detection, only the upper reference limit is reported. This comprehensive dataset provides the first serum-based pediatric RI framework encompassing organic acids, amino acids, and acylcarnitines, thereby supporting quantitative evaluation and longitudinal monitoring of metabolic disorders. The established RIs also can form the foundation for data-driven disease classification models using machine-learning approaches.

### 3.4. Clinical Validation Across Inborn Errors of Metabolism

**Figure 2:**
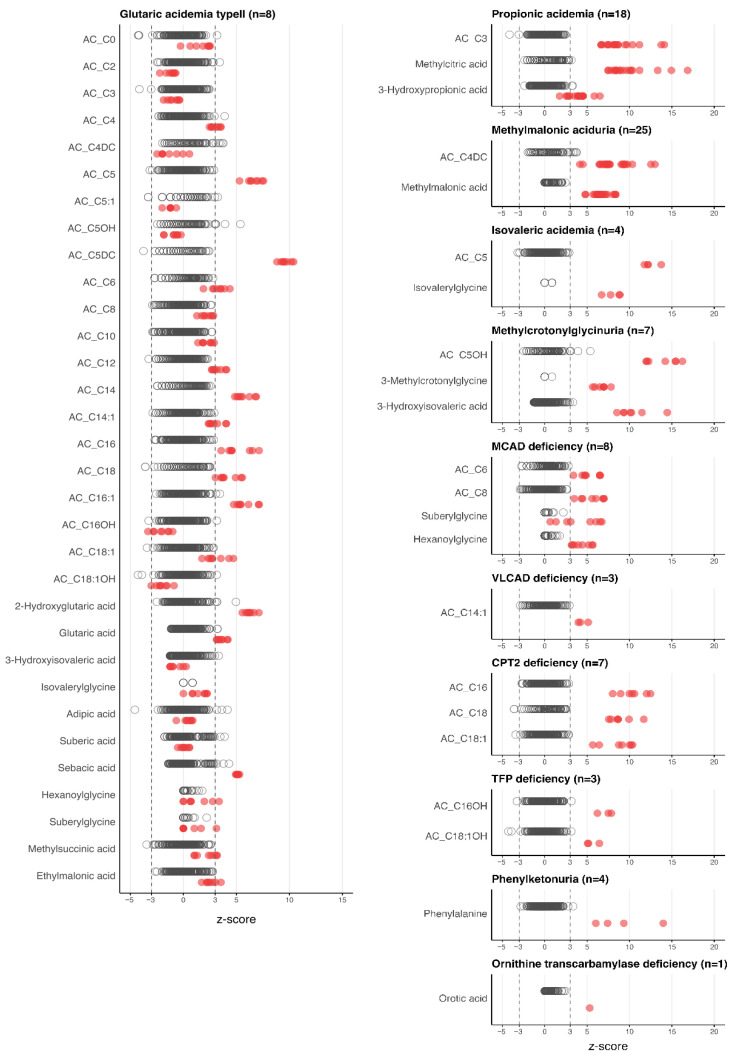
Z-scores of diagnostic markers in metabolic disorders. For each disorder, the corresponding diagnostic markers are plotted. Black circles indicate non-IEM children (*n* = 296), while red circles represent affected patients.

Clinical performance was evaluated in 89 biochemically diagnosed cases encompassing multiple IEM categories. Compared with non-IEM children, each disease group exhibited at least one metabolite with a Z-score > 3.0, indicating marked deviation from the reference distribution (Figure 2). The altered profiles corresponded well to known disease pathophysiology. For example, glutaric acidemia type II (multiple acyl-CoA dehydrogenase deficiency, OMIM #231680) exhibited elevations of short- to long-chain acylcarnitines together with organic acids such as glutaric and 2-hydroxyglutaric acid [22,23,24]. While individual quantification of these organic acids in serum is available in select clinical laboratories, the present platform enables their simultaneous and quantitative assessment together with acylcarnitines within a single serum-based analytical workflow. These findings demonstrated that disease-specific metabolic signatures can be captured quantitatively from serum in a single analytical run, including metabolites that have traditionally required urine-based testing. To explore whether these quantitative serum data could further support diagnostic discrimination, we next conducted a pilot statistical modeling analysis using machine-learning-oriented approaches.

### 3.5. Pilot Multinominal Logistic Regression Analysis for Machine-Learning-Assisted Diagnosis

As a proof-of-concept step toward data-driven diagnostics, we constructed pilot multinomial logistic regression (MLR) models using z-score-normalized values of representative diagnostic metabolites to distinguish among key IEM categories. The analyses targeted three disease group: (i) propionic acidemia (PA) and methylmalonic acidemia (MMA); (ii) isovaleric acidemia (IVA) and 3-methylcrotonyl-CoA carboxylase deficiency (MCC); (iii) long-chain fatty acid oxidation disorders, including very-long-chain acyl-CoA dehydrogenase (VLCAD), carnitine palmitoyltransferase II (CPT2), and mitochondrial trifunctional protein (TFP) deficiencies.

**Figure 3:**
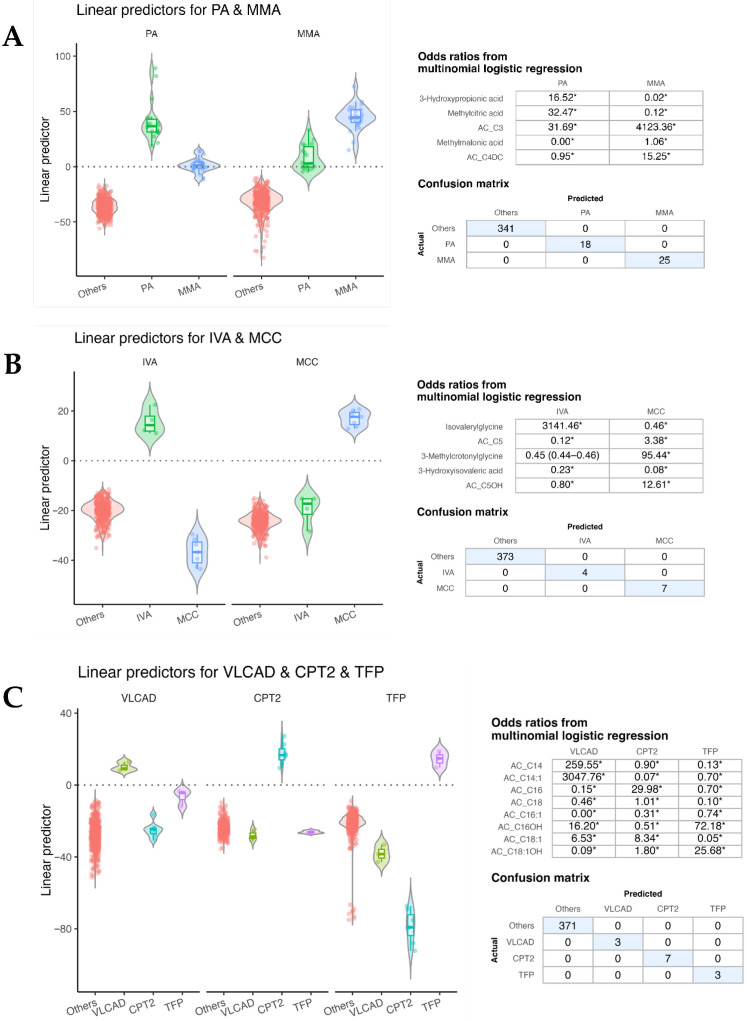
Multinomial logistic regression models for representative inborn errors of metabolism. (**A**) Propionic acidemia (PA) and methylmalonic acidemia (MMA). (**B**) Isovaleric acidemia (IVA) and 3-methylcrotonyl-CoA carboxylase deficiency (MCC). (**C**) Long-chain fatty acid oxidation disorders including very-long-chain acyl-CoA dehydrogenase (VLCAD), carnitine palmitoyltransferase II (CPT2), and mitochondrial trifunctional protein (TFP) deficiencies. For each model, the left panel shows the distribution of linear predictors for each disease versus other groups. The upper-right table summarize odds ratio derived from logistic regression for representative diagnostic metabolites. For simplicity, 95% confidence intervals were omitted and are denoted as “*”, as nearly all extended beyond the analytical range (approximately <0.01–>999) due to small sample sizes and sparse events in these rare disease cohorts. the lower-right tables show the confusion matrix comparing predicted and actual diagnostic categories.

Figure 3A shows the MLR results for differentiating PA and MMA based on serum metabolite profiles. Among the 54 quantified metabolites, five diagnostic markers—3-hydroxypropionic acid, methylcitric acid, methylmalonic acid, propionylcarnitine (AC_C3), and methylmalonylcarnitine (AC_C4DC)—were incorporated into the model. Linear predictors were calculated as weighted sums of Z-scores, reflecting each metabolite’s regression coefficient. Distinct separation of PA and MMA was observed along the predictor axis, with odds ratios confirming strong contributions of propionate-pathway metabolites. The confusion matrix demonstrated perfect classification performance, correctly identifying all PA (*n* = 18) and MMA (*n* = 25) cases without misclassification among 341 non-IEM children.

Figure 3B,C presents similar MLR frameworks applied to the other disease groups. The model for IVA and MCC accurately distinguished between these two branched-chain amino acid disorders (Figure 3B), while the model for long-chain fatty acid oxidation disorders successfully separated VLCAD, CPT2, and TFP deficiencies with clear boundaries of linear predictors (Figure 3C). Across all models, predicted classifications showed strong concordance with biochemical diagnoses, supporting the feasibility of quantitative, serum-based metabolite profiling for multivariate disease discrimination. These proof-of-concept results highlight the potential of integrating standardized metabolomic quantification with machine-learning-assisted diagnostic systems, underscoring the long-term applicability of the fully automated serum LC–MS/MS platform in precision metabolic medicine.

## 4. Discussion

We developed and validated a fully automated serum LC–MS/MS workflow that consolidates second-line testing for IEMs—including organic acids, amino acids, and acylcarnitines—into a single analytical run from one serum specimen. The total analysis time of within 60 min per sample. By integrating on-instrument sample preparation with multiplexed LC–MS/MS detection, the platform directly addresses two entrenched limitations of the current multi-assay paradigm: fragmentation of testing across distinct specimen types and platforms, and pre-analytical complexity and delays, particularly when urinary GC–MS is outsourced [12,13]. Automation minimizes operator-dependent variability, enables predictable batching, and reduces staffing burden. These advantages facilitate implementation in routine clinical laboratories, where rapid turnaround time, reproducibility, and timely availability of results are critical operational priorities, particularly when centralized reference testing would otherwise delay clinical decision-making. Together, these features position the system as a practical, high-performance framework for standardized metabolic assessment, enabling timely clinical decision-making in both acute presentations and chronic care settings.

### 4.1. Analytical and Operational Performance

From an analytical perspective, the validated linear ranges encompassed physiological concentrations for most target metabolites observed in non-IEM children (*n* = 296), allowing quantitative reporting against the newly established serum-based pediatric RIs. Despite employing single-point calibration for 34 internal-standardized analytes, imprecision was uniformly <15% CV across all 54 targets, in line with ICH M10 and FDA bioanalytical guidelines [20,21]. This trade-off between analytical complexity and operational feasibility is justified in the context of acute metabolic crises, where turnaround time often outweighs incremental precision gains from multipoint calibration. Two metabolites—3-hydroxybutyric acid and glutamine—exceeded the upper limit of linearity in some specimens, suggesting the need for future optimization of MRM parameters, extended calibration ranges, or adaptive dilution strategies.

Although the majority of analytes demonstrated acceptable analytical performance, several metabolites, including methylcitric acid, isovalerylglycine, 3-methylcrotonylglycine, AC_C6, and C18, exhibited notable systematic deviations from nominal concentrations across concentration levels. For AC_C6, the absence of a structurally matched stable isotope-labeled internal standard may have contributed to reduced quantitative accuracy. For the other metabolites, isotopically matched internal standards were used; however, residual systematic bias may arise from compound-specific analytical characteristics under multiplex conditions. In particular, for 3-methylcrotonylglycine, incomplete chromatographic resolution from the isobaric compound tiglylglycine may have contributed to the observed positive deviation. This reflects the inherent challenge of resolving structurally related isomers in a high-throughput LC–MS/MS platform. A similar consideration applies to AC_C5, where isovalerylcarnitine and 2-methylbutyrylcarnitine were evaluated using authentic reference standards. Although complete baseline separation was not achieved, a consistent difference in retention time allowed practical chromatographic differentiation under the present conditions (Supplemental Figure S1). Despite these limitations in absolute quantification for selected analytes, clinical interpretation in the present study was based on integrated disease-specific metabolic profiles rather than isolated single-analyte values. Therefore, while caution is warranted when interpreting absolute concentrations for these compounds, the overall diagnostic performance and pattern-recognition capability of the method remain robust.

Operationally, full automation standardizes pre-analytical steps such as protein precipitation and 3-NPH derivatization, reducing variability due to manual timing, temperature, or reagent handling. Consolidating testing into a single serum matrix simplifies sample routing, reduces matrix heterogeneity across assays, and facilitates integration with Laboratory Information Systems (LIS). In scenarios where urine collection is delayed or infeasible—such as in neonates [25] or critically ill patients—the single-serum workflow prevents diagnostic interruptions and ensures continuous clinical support. Although current guidelines recommend heparinized plasma for acylcarnitine analysis [11], serum was used in this study, reflecting both the availability of archived patient samples and the routine use of serum in clinical biochemical testing at our institution.

### 4.2. Clinical Significance of a Unified Serum Platform

Clinically, this enables integrated metabolic assessment using the same sample typically collected for standard biochemistry, allowing early diagnosis, follow-up, and therapy monitoring. The unified workflow supports real-time decision-making and could shorten the time between suspicion and intervention. The quantitative data obtained also permit longitudinal follow-up of disease activity, therapeutic response, and residual metabolic load, transforming static diagnostic testing into dynamic clinical monitoring.

### 4.3. Establishment and Interpretation of Pediatric RIs

A major strength of this study is the establishment of serum-based pediatric RIs for 54 metabolites across 0–6 years of age. Given the strong age dependence of metabolic fluxes—driven by growth, nutrition, and enzyme maturation—age-adjusted RIs are essential for accurate interpretation. The parametric Box–Cox framework with outlier exclusion (±2.81 SD) and back-transformation (±1.96 SD) provided clinically interpretable limits even for skewed distributions. For analytes with >2.5% of measurements below the detection limit, only upper limits were defined, reflecting empirical detectability in pediatric serum.

Our serum RIs complement existing plasma-based catalogs [26,27,28] and address known matrix-dependent differences. For instance, several amino acids exhibit higher concentrations in serum than plasma [29,30]. Thus, serum-specific RIs provide practical interpretive value for laboratories operating primarily on serum workflows. We also compiled previously reported plasma-based reference data alongside our serum results for comparison (Supplemental Table S6), facilitating inter-laboratory harmonization. The availability of these RIs enables quantitative interpretation of metabolite deviations, transforming conventional qualitative pattern recognition into standardized, quantitative disease assessment.

While the Clinical and Laboratory Standards Institute (CLSI) EP28-A3c guideline [31] is widely recognized as the primary reference for establishing RIs, we adopted the IFCC C-RIDL protocol to ensure alignment with international harmonization initiatives specifically designed for multicenter, population-based studies [19,32]. The C-RIDL approach allows the use of parametric normalization (e.g., Box–Cox or logarithmic transformation) and supports direct comparison of datasets across institutions and ethnicities, which is particularly important for pediatric cohorts where large sample sizes are difficult to obtain at a single site. Moreover, the C-RIDL framework has been successfully applied in Asian pediatric populations and provides a validated pathway for indirect transfer and harmonization of RIs [32]. Therefore, although not the standard CLSI pathway, the IFCC C-RIDL methodology was intentionally selected to ensure global comparability and reproducibility of pediatric reference data.

### 4.4. Quantitative Disease Evaluation and Machine-Learning-Assisted Modeling

Clinical validation in 89 biochemically confirmed IEM cases demonstrated that disease-specific metabolic patterns can be captured directly from serum, even for metabolites currently measured in urine. Representative examples include glutaric acidemia type II, characterized by elevated organic acids (glutaric, 2-hydroxyglutaric) and broad acylcarnitine accumulation [22,23,24], and propionic and methylmalonic acidemias, which exhibit disease-specific combinations of methylcitric acid, propionylcarnitine (AC_C3), and methylmalonic acid reflecting the site of enzymatic blockage [33,34]. In the present study, the MMA cases were included as biochemically confirmed methylmalonic aciduria based on elevated methylmalonic acid levels; however, detailed subclassification (e.g., mut, cblA/B versus cblC-related combined MMA) was not available for all archived samples. In patients with propionic acidemia (PA) and methylmalonic acidemia (MMA), plasma-based analyses have shown that related organic acids can be directly quantified and that their concentrations are associated with disease status and metabolic control [35,36]. These observations were consistent with known biochemical pathways and confirm the diagnostic fidelity of serum-based analysis.

To explore the potential of data-driven interpretation, we applied pilot multinomial logistic regression models using z-score–normalized metabolite data derived from the pediatric RIs. The models successfully discriminated key IEM categories: propionic/methylmalonic acidemias, isovaleric/3-methylcrotonyl-CoA carboxylase deficiencies (IVA/MCC), and long-chain fatty acid oxidation disorders (VLCAD, CPT2, TFP deficiencies). Among the predefined markers, methylmalonic acid and acylcarnitine C4DC were more strongly associated with MMA, whereas 3-hydroxypropionic acid, methylcitric acid, and C3-carnitine showed higher odds ratios in PA—findings concordant with established biochemical distinctions described in consensus guidelines [33]. Although exploratory, these results demonstrate that quantitative serum data preserve diagnostic information suitable for multivariate, machine-learning-assisted classification.

While further work is needed to expand model size and diversity, this proof-of-concept underscores the feasibility of combining standardized quantitative metabolite data with statistical modeling to improve reproducibility and objectivity in IEM diagnostics. In the long term, integration of automated LC–MS/MS workflows with machine-learning-assisted interpretation could facilitate semi-automated diagnostic support in routine practice, analogous to algorithmic decision systems in clinical chemistry or hematology.

### 4.5. Limitation and Future Directions

This study has several limitations. The number of available cases for each IEM category was relatively small, limiting statistical power and exploration of rarer phenotypes. Commercial internal standards were available for 34 of the 54 target analytes; for the remaining compounds, their unavailability necessitated semi-quantitative assessment and may have affected cross-lot comparability. Another analytical limitation relates to the chromatographic resolution of certain isobaric amino acids. Under the multiplex LC–MS/MS conditions used in this study, complete chromatographic separation between leucine and isoleucine was not achieved, and hydroxyproline, which is also isobaric with leucine, was not specifically evaluated as an independent analyte. However, in human serum leucine is typically present at substantially higher concentrations than hydroxyproline, and leucine and isoleucine are generally present in comparable ranges and biologically correlated as branched-chain amino acids. Because the present study focuses on relative metabolite quantification within a uniformly processed sample set rather than diagnostic discrimination of individual branched-chain amino acids, this limitation is unlikely to substantially affect the comparative analyses performed in this work. Nevertheless, applications requiring strict discrimination among leucine, isoleucine, and hydroxyproline would require dedicated chromatographic separation.

Another limitation is that total homocysteine (tHcy), an essential marker for the differential diagnosis between isolated methylmalonic acidemia and combined MMA with homocystinuria, was not included in the present analyte panel. In clinical practice, diagnosis of MMA typically follows a stepwise biochemical algorithm: detection of elevated methylmalonic acid during initial metabolic screening, followed by targeted measurement of tHcy to distinguish isolated MMA from combined cobalamin-related disorders, and subsequent confirmation by molecular genetic testing. Therefore, the present multiplex metabolite platform is intended to function primarily as a biochemical screening and metabolic monitoring tool within this established diagnostic workflow rather than as a stand-alone assay for subtype discrimination.

In addition, most patient samples analyzed in this study were archived specimens obtained from external institutions, and detailed clinical information regarding treatment status or sampling time points was not consistently available. Therefore, the present dataset does not allow direct evaluation of longitudinal metabolite changes during therapy. Prospective studies with well-defined pre- and post-treatment sampling will be required to formally validate the clinical utility of this platform for therapeutic monitoring.

The current panel does not include certain newborn-screening-relevant metabolites such as argininosuccinic acid and succinylacetone [37,38]. In several specimens, concentrations of 3-hydroxybutyric acid and glutamine exceeded the validated upper limit of linearity. These metabolites are known to increase markedly during acute metabolic decompensations such as ketosis or hyperammonemic crises in patients with inborn errors of metabolism. In such cases, dilution and re-analysis can be readily implemented within the current LC–MS/MS workflow, and future methodological refinements will aim to extend the calibration range to improve quantitative performance at very high concentrations. Although our pediatric cohort was relatively large, RIs were not partitioned by narrow age bands or sex, and neonatal time points (e.g., postnatal day 3–5)—which correspond to the standard sampling window for newborn screening [39]—were not represented. Finally, inter-laboratory comparisons and participation in external quality assessment (EQA) schemes were not performed but will be essential for broader validation and proficiency alignment. External validation through international collaborative studies will be essential to generalize these RIs beyond the Japanese population.

Future studies should aim to expand the analyte library, optimize analytical ranges, and establish age- and sex-partitioned RIs, including neonatal subsets. Collaborative, multi-institutional efforts combining clinical, biochemical, and genetic data will be crucial to build robust machine-learning-assisted models and validate them across diverse populations. Further integration into laboratory information systems may support automated flagging, trend analysis, and harmonized reporting across metabolic centers.

In summary, this study provides a foundation for serum-based, quantitative, and fully automated assessment of inborn errors of metabolism. By defining pediatric RIs and demonstrating proof-of-concept machine-learning-assisted classification, we show that serum metabolomics can unify second-line testing, shorten diagnostic turnaround, and enable data-driven interpretation. This platform represents a practical step toward precision metabolic diagnostics that are standardized, reproducible, and operationally feasible for modern clinical laboratories.

## Figures and Tables

**Table 1:** Linearity and sensitivity.

Component	IS	Calibration Range (µmol/L)		Calibration Points	r^2^	LOD (µmol/L)	LOQ (µmol/L)
		Lower	Upper				
3-Hydroxypropionic acid	^13^C_3__3-Hydroxypropionic acid	0.20	100	10	0.9997	0.391	1.563
Methylcitric acid	^2^H_3__Methylcitric acid	0.20	50	9	0.9973	0.024	0.098
Methylmalonic acid	^13^C_4__Methylmalonic acid	0.20	100	10	0.9996	0.195	0.391
Lactic acid	^13^C_3__Lactic acid	39.06	20,000	10	0.9989	19.531	78.125
Pyruvic acid	^13^C_3__Pyruvic acid	3.91	2000	10	0.9997	0.977	7.813
3-Hydroxyisovaleric acid	^2^H_8__3-Hydroxyisovaleric acid	0.39	50	8	0.9959	1.563	6.250
Isovalerylglycine	^13^C_2__^15^N_1__Isovalerylglycine	0.20	50	9	0.9999	0.012	0.049
3-Methylcrotonylglycine	^13^C_2__^15^N_1__3-Methylcrotonylglycine	0.20	25	8	0.9990	0.006	0.012
3-Methylglutaric acid	-	0.20	25	8	0.9979	0.049	0.195
3-Methylglutaconic acid	-	0.20	25	8	0.9978	0.049	0.098
3-Hydroxy-3-methylglutaric acid	-	0.20	25	8	0.9994	0.049	0.195
3-Hydroxyglutaric acid	^2^H_5__3-Hydoroxyglutaric acid	0.20	100	10	0.9992	0.024	0.098
2-Hydroxyglutaric acid	-	0.20	50	9	0.9963	0.024	0.098
Glutaric acid	^13^C_2__Glutaric acid	0.20	25	8	0.9930	0.195	1.563
2-Methyl-3-hydroxybutyric acid	-	0.20	50	9	0.9989	0.012	1.563
Tiglylglycine	-	0.20	25	8	0.9995	0.006	0.006
Orotic acid	-	0.20	25	8	0.9967	0.078	0.078
3-Hydroxybutyric acid	-	0.98	125	8	0.9932	0.122	0.488
Adipic acid	-	0.20	25	8	0.9989	0.391	1.563
Suberic acid	-	0.20	25	8	0.9981	0.195	0.781
Sebacic acid	-	0.20	25	8	0.9971	0.098	0.781
Hexanoylglycine	-	0.20	25	8	0.9983	0.006	0.006
Suberylglycine	-	0.20	25	8	0.9956	0.006	0.049
Methylsuccinic acid	-	0.20	25	8	0.9956	0.781	6.250
Ethylmalonic acid	-	0.20	25	8	0.9989	0.012	0.049
Arginine	^13^C_6__Arginine	3.91	500	8	0.9999	1.953	1.953
Glutamine	-	3.91	1000	9	0.9912	0.488	0.977
Citrulline	^2^H_7__Citrulline	1.95	500	9	0.9999	0.031	0.122
Valine	^2^H_7__Valine	1.95	500	9	0.9978	1.953	15.625
Tyrosine	^13^C_6__Tyrosine	1.95	500	9	0.9987	0.488	1.953
Methionine	^2^H_3__Methionine	1.95	500	9	0.9993	0.244	0.244
Leucine	^2^H_3__Leucine	1.95	500	9	0.9991	7.813	31.250
Phenylalanine	^13^C_6__Phenylalanine	1.95	500	9	0.9996	0.122	0.977
AC_C0	^2^H_9__AC_C0	0.20	100	10	0.9997	0.0244	0.0488
AC_C2	^2^H_3__AC_C2	0.10	50	10	0.9994	0.0031	0.0061
AC_C3	^2^H_3__AC_C3	0.02	12	10	0.9994	0.0015	0.0015
AC_C4	^2^H_3__AC_C4	0.01	5	10	0.9998	0.0024	0.0098
AC_C4DC	^2^H_9__AC_C5DC	0.01	5	10	0.9997	0.0003	0.0006
AC_C5	^2^H_9__AC_C5	0.01	3	10	1.0000	0.0002	0.0007
AC_C5:1	^2^H_9__AC_C5	0.01	5	10	0.9992	0.0003	0.0003
AC_C5OH	^2^H_9__AC_C5OH	0.01	5	10	0.9993	0.0006	0.0006
AC_C5DC	^2^H_9__AC_C5DC	0.00	2.5	10	0.9993	0.0003	0.0006
AC_C6	^2^H_3__AC_C8	0.00	1.25	9	0.9992	0.0003	0.0024
AC_C8	^2^H_3__AC_C8	0.00	2.5	10	0.9996	0.0002	0.0003
AC_C10	^2^H_3__AC_C10	0.00	2.5	10	0.9999	0.0003	0.0012
AC_C12	^2^H_3__AC_C12	0.00	2.5	10	0.9998	0.0003	0.0012
AC_C14	^2^H_3__AC_C14	0.01	3	10	0.9998	0.0007	0.0029
AC_C14:1	^2^H_3__AC_C14:1	0.01	3	10	0.9994	0.0007	0.0029
AC_C16	^2^H_3__AC_C16	0.02	12	10	0.9999	0.0059	0.0234
AC_C18	^2^H_3__AC_C18	0.01	5	10	0.9996	0.0003	0.0195
AC_C16:1	-	-	-	-	-	-	-
AC_C16OH	-	-	-	-	-	-	-
AC_C18:1	-	-	-	-	-	-	-
AC_C18:1OH	-	-	-	-	-	-	-

**Table 2:** Accuracy and imprecision.

Compound Name	Units	LQC					MQC					HQC				
			Within-Run		Between-Run			Within-Run		Between-Run			Within-Run		Between-Run	
		Nominal Conc	Accu (%)	CV%	Accu (%)	CV%	Nominal Conc	Accu (%)	CV%	Accu (%)	CV%	Nominal Conc	Accu (%)	CV%	Accu (%)	CV%
3-Hydroxypropionic acid	µmol/L	2.69	109.4	1.9	108.5	4.0	6.44	117.6	1.5	119.8	3.0	21.44	110.4	0.6	112.7	3.0
Methylcitric acid	µmol/L	1.33	166.3	4.3	175.6	3.2	5.08	175.0	2.3	176.4	2.6	20.08	137.4	0.7	142.9	3.2
Methylmalonic acid	µmol/L	1.39	99.0	2.0	98.4	4.3	5.14	99.0	1.9	100.8	5.2	20.14	86.4	2.1	88.6	1.2
Lactic acid	µmol/L	1504.4	97.4	1.8	99.7	4.3	2254.4	89.4	1.4	91.9	5.9	5254.4	76.0	1.0	77.3	3.2
Pyruvic acid	µmol/L	124.5	108.9	1.0	113.3	3.3	199.5	128.3	0.8	133.2	3.7	499.5	135.4	0.2	139.5	3.4
3OH isovaleric acid	µmol/L	2.87	80.6	2.3	81.6	9.3	6.62	84.3	3.9	85.9	5.3	21.62	68.7	2.5	69.2	3.9
Isovalerylglycine	µmol/L	1.25	71.9	2.5	72.1	2.7	5.00	71.5	4.7	72.6	6.6	20.00	64.5	1.3	64.8	2.6
3-Methylcrotonylglycine	µmol/L	1.25	210.1	4.1	219.2	3.1	5.00	202.7	2.4	214.6	5.4	20.00	184.7	3.7	184.7	1.8
3-Methylglutaric acid	a.u.	26,290	-	3.1	-	10.1	26,293	-	0.6	-	4.3	26,308	-	0.7	-	4.8
3-Methylglutaconic acid	a.u.	47,868	-	4.7	-	7.1	47,872	-	2.1	-	5.8	47,887	-	1.3	-	3.5
3-Hydroxy-3-methylglutaric acid	a.u.	99,323	-	2.3	-	7.2	99,327	-	1.6	-	4.6	99,342	-	2.3	-	2.8
3-Hydroxyglutaric acid	µmol/L	1.30	94.4	1.5	97.6	3.0	5.05	93.8	2.2	97.4	5.7	20.05	84.8	2.2	87.0	2.4
2-Hydroxyglutaric acid	a.u.	124,371	-	2.8	-	9.1	124,375	-	0.7	-	7.1	124,390	-	3.6	-	7.2
Glutaric acid	µmol/L	1.45	104.2	2.6	108.8	4.6	5.20	99.9	2.0	105.0	4.1	20.20	75.6	2.5	78.2	1.1
2-Methyl-3-hydroxybutyric acid	a.u.	94,449	-	1.5	-	8.8	94,453	-	0.9	-	4.5	94,468	-	1.3	-	4.7
Tiglylglycine	a.u.	305	-	1.5	-	5.3	308	-	2.3	-	3.8	323	-	0.7	-	2.9
Orotic acid	a.u.	1201	-	8.4	-	12.9	1202	-	11.2	-	12.4	1205	-	5.4	-	8.8
3-Hydroxybutyric acid	a.u.	2,210,297	-	0.8	-	4.1	2,210,316	-	1.7	-	3.8	2,210,391	-	1.7	-	4.0
Adipic acid	a.u.	28,731	-	2.3	-	9.2	28,734	-	0.9	-	6.6	28,749	-	0.5	-	5.7
Suberic acid	a.u.	7946	-	2.6	-	9.0	7950	-	2.3	-	6.0	7965	-	1.1	-	4.5
Sebacic acid	a.u.	7055	-	2.9	-	7.4	7058	-	4.2	-	4.2	7073	-	1.3	-	3.9
Hexanoylglycine	a.u.	828	-	0.9	-	12.1	832	-	1.7	-	6.9	847	-	2.2	-	9.7
Suberylglycine	a.u.	682	-	1.5	-	5.7	686	-	2.0	-	4.4	701	-	1.6	-	3.9
MethylsuccinicAcid	a.u.	98,260	-	6.3	-	9.6	98,264	-	1.4	-	5.6	98,279	-	2.2	-	3.9
EthylmalonicAcid	a.u.	60,420	-	4.6	-	2.8	60,423	-	2.3	-	7.4	60,438	-	2.8	-	6.0
Arginine	µmol/L	125.6	98.7	1.8	127.7	14.0	144.4	101.4	7.0	124.2	14.4	219.4	103.8	1.2	118.1	12.5
Glutamine	a.u.	1,803,614	-	4.4	-	14.6	1,803,689	-	7.8	-	4.5	1,803,989	-	3.6	-	7.0
Citrulline	µmol/L	35.2	106.6	2.8	108.2	1.7	53.9	117.5	1.0	118.4	1.7	128.9	115.6	0.7	117.4	1.7
Valine	µmol/L	160.7	91.8	3.6	101.3	3.3	198.2	94.6	1.8	101.1	6.1	348.2	89.0	4.9	88.6	3.0
Tyrosine	µmol/L	79.7	101.6	2.0	104.9	2.7	98.5	116.1	1.8	116.1	2.6	173.5	115.8	3.3	124.8	5.5
Methionine	µmol/L	27.8	104.8	2.9	111.2	5.9	46.5	118.6	5.2	118.8	7.3	121.5	114.9	4.0	116.7	7.3
Leucine	µmol/L	120.3	104.5	1.5	115.4	5.2	139.0	107.4	2.5	118.8	8.9	214.0	109.7	2.0	115.5	6.6
Phenylalanine	µmol/L	55.1	101.8	1.7	111.8	7.2	73.9	104.8	1.5	115.2	7.0	148.9	104.4	1.4	109.8	7.2
AC_C0	µmol/L	15.59	97.6	2.7	101.2	7.6	19.34	95.0	3.3	96.6	6.8	34.34	84.9	2.1	82.4	5.5
AC_C2	µmol/L	5.08	101.4	1.2	103.5	1.4	6.96	101.6	0.9	106.2	3.5	14.46	96.7	0.5	101.0	3.8
AC_C3	µmol/L	0.362	103.7	2.6	102.1	2.7	0.812	105.9	1.4	106.3	0.9	2.612	92.5	1.8	93.5	3.6
AC_C4	µmol/L	0.143	99.4	3.1	98.9	4.9	0.331	101.7	3.3	102.5	2.8	1.081	92.2	1.5	92.8	2.6
AC_C4DC	µmol/L	0.082	87.9	1.7	86.1	5.5	0.270	86.4	2.3	84.4	4.1	1.020	76.9	1.3	76.0	2.8
AC_C5	µmol/L	0.108	105.0	2.2	104.4	2.5	0.220	103.1	1.6	108.3	2.7	0.670	93.5	1.5	97.7	2.4
AC_C5_1	µmol/L	0.069	105.2	2.3	98.1	8.9	0.257	106.0	1.8	103.8	5.5	1.007	95.1	1.7	92.5	5.8
AC_C5OH	µmol/L	0.080	118.5	2.7	118.7	5.8	0.267	129.2	1.1	130.9	4.6	1.017	117.4	1.7	118.7	3.2
AC_C5DC	µmol/L	0.048	106.4	1.9	106.5	2.7	0.142	112.7	2.2	112.9	3.1	0.517	100.6	2.7	103.7	3.4
AC_C6	µmol/L	0.053	56.5	8.9	54.2	5.1	0.146	51.2	11.4	50.7	13.7	0.521	34.9	9.2	38.9	10.9
AC_C8	µmol/L	0.109	96.8	1.5	97.4	1.3	0.202	95.0	1.5	98.0	3.2	0.577	83.4	1.3	87.4	3.4
AC_C10	µmol/L	0.210	93.8	2.9	95.9	5.4	0.303	97.4	1.0	100.0	5.6	0.678	93.0	0.7	96.0	3.4
AC_C12	µmol/L	0.067	99.4	1.0	101.1	3.2	0.160	101.5	0.7	103.3	3.1	0.535	88.1	0.8	91.6	2.9
AC_C14	µmol/L	0.049	100.4	1.1	101.7	2.8	0.162	101.7	1.0	103.5	4.2	0.612	90.5	1.0	93.2	3.7
AC_C14_1	µmol/L	0.079	101.7	1.3	103.3	3.5	0.191	90.2	1.4	93.1	3.8	0.641	76.4	0.5	79.5	3.8
AC_C16	µmol/L	0.214	91.8	1.6	93.6	4.2	0.664	90.9	0.9	94.3	3.9	2.464	87.4	0.9	90.6	3.7
AC_C18	µmol/L	0.082	56.3	1.3	55.8	1.2	0.270	50.6	1.3	51.6	2.1	1.020	71.0	0.6	73.9	3.3
AC_C16_1	a.u.	273,568	-	4.1	-	10.4	273,568	-	-	-	-	273,568	-	-	-	-
AC_C16OH	a.u.	34,793	-	4.4	-	11.3	34,793	-	-	-	-	34,793	-	-	-	-
AC_C18_1	a.u.	2,603,471	-	1.2	-	10.1	2,603,471	-	-	-	-	2,603,471	-	-	-	-
AC_C18_1OH	a.u.	66,232	-	3.3	-	12.2	66,232	-	-	-	-	66,232	-	-	-	-

**Table 3:** Reference intervals for 54 serum analytes in children aged 0–6 years.

Compound Name	Units	Reference Intervals
3-Hydroxypropionic acid	µmol/L	1.42–9.83
Methylcitric acid	µmol/L	0.10–0.38
Methylmalonic acid	µmol/L	≤0.37
Lactic acid	µmol/L	1054.4–3902.9
Pyruvic acid	µmol/L	45.4–248.4
3-Hydroxyisovaleric acid	µmol/L	≤7.03
Isovalerylglycine	µmol/L	≤0.01
3-Methylcrotonylglycine	µmol/L	≤0.01
3-Methylglutaric.acid	a.u.	13,931–57,602
3-Methylglutaconic acid	a.u.	36,298–131,339
3-Hydroxy-3-methylglutaric acid	a.u.	108,861–625,203
3-Hydroxyglutaric acid	µmol/L	≤0.45
2-Hydroxyglutaric acid	a.u.	130,861–578,833
Glutaric acid	µmol/L	≤2.10
2-Methyl-3-hydroxybutyric acid	a.u.	62,577–333,570
Tiglylglycine	a.u.	≤2334
Orotic acid	a.u.	≤3386
3-Hydroxybutyric acid	a.u.	866,586–48,966,989
Adipic acid	a.u.	14,869–165,654
Suberic acid	a.u.	≤590,676
Sebacic acid	a.u.	≤489,304
Hexanoylglycine	a.u.	≤2164
Suberylglycine	a.u.	≤2011
Methylsuccinic acid	a.u.	23,428–108,437
Ethylmalonic acid	a.u.	44,656–349,780
Arginine	µmol/L	94.4–194.1
Glutamine	a.u.	1,417,957–4,570,111
Citrulline	µmol/L	18.9–50.3
Valine	µmol/L	109.1–272.8
Tyrosine	µmol/L	56.6–160.8
Methionine	µmol/L	15.7–48.8
Leucine	µmol/L	99.6–246.5
Phenylalanine	µmol/L	55.9–114.9
AC_C0	µmol/L	13.37–28.83
AC_C2	µmol/L	3.06–14.08
AC_C3	µmol/L	0.160–0.615
AC_C4	µmol/L	0.071–0.288
AC_C4DC	µmol/L	0.017–0.051
AC_C5	µmol/L	0.035–0.163
AC_C5:1	µmol/L	0.002–0.015
AC_C5OH	µmol/L	0.011–0.041
AC_C5DC	µmol/L	0.008–0.028
AC_C6	µmol/L	0.003–0.043
AC_C8	µmol/L	0.014–0.167
AC_C10	µmol/L	0.023–0.353
AC_C12	µmol/L	0.007–0.110
AC_C14	µmol/L	0.005–0.039
AC_C14:1	µmol/L	0.008–0.130
AC_C16	µmol/L	0.040–0.126
AC_C18	µmol/L	0.011–0.038
AC_C16:1	a.u.	100,743–723,762
AC_C16OH	a.u.	20,053–107,215
AC_C18:1	a.u.	1,189,675–4,733,385
AC_C18:1OH	a.u.	18,202–209,737

## Data Availability

The data that support the findings of this study are available on request from the corresponding author. The data are not publicly available due to privacy or ethical restrictions.

## Supplementary Materials

The following supporting information can be downloaded at: https://www.mdpi.com/article/10.3390/diagnostics16060911/s1, Table S1. Standards and ISTD. Table S2. Demographic characteristics (age and sex distribution) of children without inborn errors of metabolism. Table S3. Distribution of IEM Patient cohort. Table S4. Multiple reaction monitoring transitions of 3-Nitrophenylhydrazine(3-NPH) derivatized compounds. Table S5. Summary statistics of metabolite concentrations in children without inborn errors of metabolism. Table S6. Serum pediatric RIs (0–6 years) derived in this study versus published plasma reference limits across targeted metabolites. Table S7. Inborn error s of metabolism included in current newborn metabolic screening programs in Japan. Figure S1 Representative MRM chromatograms of isovalerylcarnitine and 2-methylbutyrylcarnitine. References [40,41,42,43,44,45,46] are cited in the supplementary materials.
