# Supplementary material for: Fully Automated Serum LC-MS/MS Platform and Pediatric Reference Intervals for Organic Acids, Amino Acids, and Acylcarnitines in Children (Ages 0–6 Years): Toward Quantitative Diagnosis of Inborn Errors of Metabolism
Source: Diagnostics (Basel). 2026 Mar 19;16(6):911. doi: 10.3390/diagnostics16060911 (PMC13025015; doi:10.3390/diagnostics16060911)
Supplement: Supplementary file 1 [file diagnostics-16-00911-s001.zip › Supplemental Figure S1 .pdf]

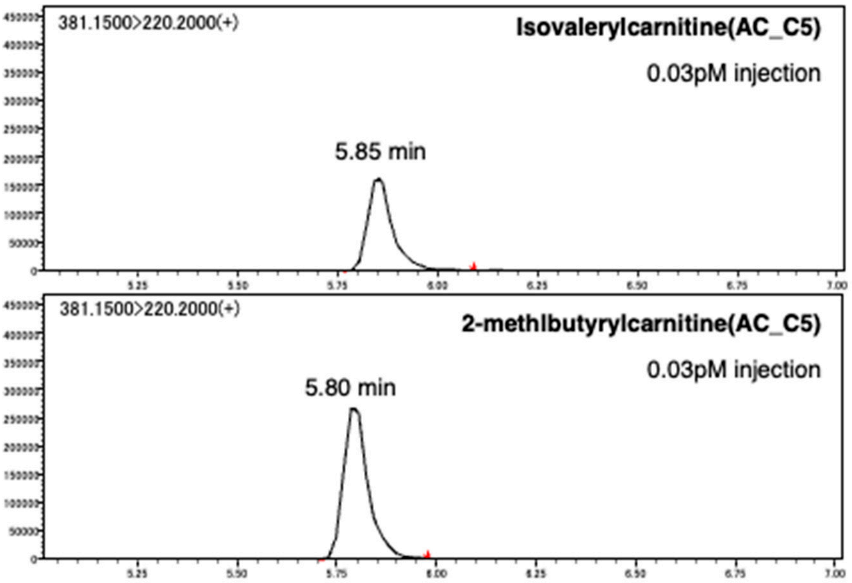

Supplemental Figure S1 Representative MRM chromatograms of isovalerylcarnitine and 2-methylbutyrylcarnitine.

Authentic standards of isovalerylcarnitine (upper panel) and 2-methylbutyrylcarnitine (lower panel) were analyzed within the same batch under the present analytical conditions. Although complete baseline separation was not achieved, the peak apices were consistently separated by approximately 0.05 min, allowing practical chromatographic differentiation of these isomeric C5-acylcarnitines.
